# Oral SARS‐CoV‐2 Infection and Risk for Long Covid

**DOI:** 10.1002/rmv.70029

**Published:** 2025-03-12

**Authors:** Joel Schwartz, Kristelle Capistrano, Heba Hussein, Avin Hafedi, Deepak Shukla, Afsar Naqvi

**Affiliations:** ^1^ Department of Oral Medicine and Diagnostic Sciences University of Illinois Chicago Chicago Illinois USA; ^2^ Department of Periodontics University of Illinois Chicago Chicago Illinois USA; ^3^ Department of Oral Medicine Faculty of Dentistry Cairo University Cairo Egypt; ^4^ Department of Microbiology and Immunology University of Illinois Chicago Chicago Illinois USA; ^5^ Department of Ophthalmology and Visual Sciences University of Illinois Chicago Chicago Illinois USA

**Keywords:** long Covid, oral microbiome, oral mucosal immunity, SARS‐CoV‐2, viral clearance

## Abstract

SARS‐CoV‐2 is an oral pathogen that infects and replicates in mucosal and salivary epithelial cells, contributing to oral post‐acute sequelae COVID‐19 (PASC) and other oral and non‐oral pathologies. While pre‐existing inflammatory oral diseases provides a conducive environment for the virus, acute infection and persistence of SARS‐CoV‐2 can also results in oral microbiome dysbiosis that further worsens poor oral mucosal health. Indeed, oral PASC includes periodontal diseases, dysgeusia, xerostomia, pharyngitis, oral keratoses, and pulpitis suggesting significant bacterial contributions to SARS‐CoV‐2 and oral tissue tropism. Dysbiotic microbiome‐induced inflammation can promote viral entry via angiotensin‐converting enzyme receptor‐2 (ACE2), serine transmembrane TMPRSS2 and possibly other non‐canonical pathways. Additionally, metabolites derived from a dysbiotic microbiome can alter the physiological and biochemical pathways related to the metabolism of lipids, carbohydrates, and amino acids. This may promote a pro‐inflammatory microenvironment, leading to immune exhaustion, loss of tolerance, and susceptibility to a variety of oral pathogens, causing acute and later chronic inflammation. Microbial release of mimics of host metallopeptidases related to furin, ADAM17 (A disintegrin and metalloproteinase 17), and glycoprotein metabolites can further aid viral attachment to T cell immunoglobulin‐like (TIMs), enhancing viral entry while simultaneously depressing oral mucosal immune resistance and clearance. Membrane reorganization characterised by neuroproteins, such as neuropilins, also functionally assists with SARS‐CoV‐2 entry and extends the pathogenesis of PASC from the oral cavity to the brain, gut, or other non‐oral tissues. Thus, poor oral health, characterised by disrupted oral microbiomes can promote viral tropism, weaken antiviral resistance, and heightens susceptibility to SARS‐CoV‐2 infection. This immune dysfunction also increases the risk of additional viral infections, exacerbating oral conditions like periodontal and endodontic diseases. These persistent oral health issues can contribute to systemic inflammation, creating bidirectional effects between oral and non‐oral tissues, potentially leading to Post‐Acute Sequelae of COVID‐19 (PASC).

**Abbreviations**
ACE2: Angiotensin‐Converting Enzyme 2
ADAM: A Disintegrin and Metalloproteinase
AGE: Advanced Glycation End‐Products
AMP: Antimicrobial Peptide
AMP: Antimicrobial Peptides
AMPs: Antimicrobial Peptides
ArhR: Aryl Hydrocarbon Receptor
ATP: Adenosine Triphosphate
CD: Cluster of Differentiation
CD73: Cluster of Differentiation 73
CD80/CD86: Cluster of Differentiation 80/86
CFS: Chronic Fatigue Syndrome
CTLA‐4: Cytotoxic T‐Lymphocyte‐Associated Protein 4
DAMP: Damage‐Associated Molecular Pattern
DC: Dendritic Cell
ETS‐1: Erythroblast Transformation‐Specific‐1
FAS/FASL: Fas Receptor/Fas Ligand
FICZ: 6‐Formylindolo[3,2‐b]carbazole
Foxp3: Forkhead Box P3
GCF: Gingival Crevicular Fluid
HIF‐1: Hypoxia‐Inducible Factor 1
IFN: Interferon
IL: Interleukin
iNOS: Inducible Nitric Oxide Synthase
IRF: Interferon Regulatory Factors
iTreg: Induced Regulatory T Cells
MHC: Major Histocompatibility Complex
MHC II: Major Histocompatibility Complex Class II
MMP: Matrix Metalloproteinases
mTOR: Mammalian Target of Rapamycin
NEAA: Non‐Essential Amino Acids
NFAT: Nuclear Factor of Activated T Cells
NK: Natural Killer Cells
NO: Nitric Oxide
Nrf2: Nuclear Factor Erythroid 2‐Related Factor 2
PAMP: Pathogen‐Associated Molecular Pattern
PASC: Post‐Acute Sequelae of COVID‐19
PD‐1: Programed Death‐1
PGE2: Prostaglandin E2
POTs: Postural Orthostatic Tachycardia Syndrome
PPAR: Peroxisome Proliferator‐Activated Receptor
PPR: Pathogen Pattern Recognition
PRR: Pattern Recognition Receptor
PTEN: Phosphatase and Tensin Homologue
RAGE: Receptor for Advanced Glycation End‐Products
ROS: Reactive Oxygen Species
S protein: Spike Protein
SARS‐CoV‐2: Severe Acute Respiratory Syndrome Coronavirus 2
SNARE: Soluble N‐Ethylmaleimide‐Sensitive Factor Attachment Protein Receptor
STAT: Signal Transducer and Activator of Transcription
TGF‐β: Transforming Growth Factor Beta
Th1/Th17: T Helper 1/T Helper 17 Cells
TIM: T Cell Immunoglobulin‐Like Receptors
TLR: Toll‐Like Receptor
TMPRSS: Transmembrane Serine Protease
TRPA1: Transient Receptor Potential Ankyrin 1

## 1 Introduction

While widespread vaccination and public health efforts have alleviated the global impact of COVID‐19 compared to the earlier stages of the pandemic, SARS‐CoV‐2 remains a persistent threat. The recent emergence of new variants, notably KP.3.1.1, and fluctuating outbreaks continue to render vulnerable individuals susceptible to COVID‐19‐related hospitalisation and death, highlighting the ongoing need for vigilance [1]. As of August 2024, test positivity rates in the United States hover around 18%, COVID‐19‐related emergency department visits continue to rise, and wastewater surveillance for SARS‐CoV‐2, indicating high viral levels across most regions in the country. All these pieces of evidence suggest that the virus will likely remain a long‐term presence. Supporting SARS‐CoV‐2 transmission and longevity is the oral mucosa, which recent evidence implicates as a significant site of viral activity. SARS‐CoV‐2 can bind to oral host cell receptors, mainly angiotensin‐converting enzyme 2 (ACE2) and transmembrane serine proteases (TMPRSS), expressed on the surface of the cells and the mucosal lining, allowing the oral cavity to act as a portal of entry for the virus [2]. Beyond serving as an entry point for the virus, the oral cavity serves as a conducive environment where the virus can evade the host immune response, replicate and persist [2, 3].

**FIGURE 1:**
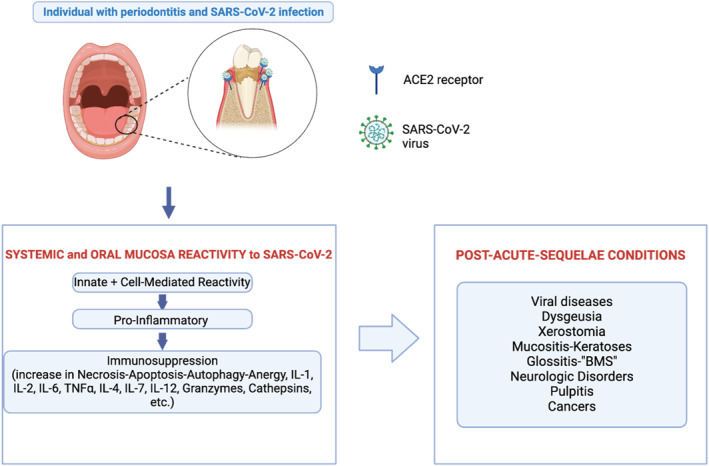
Chronic inflammation associated with periodontitis compromises the local immune defenses in the oral cavity. This pre‐existing inflammatory state in periodontitis is exacerbated by SARS‐CoV‐2 infection, which overstimulates the immune response, causing excessive inflammation. Over time, this heightened inflammation leads to immune exhaustion and subsequent immunosuppression, reducing the body's ability to eliminate the virus and enabling its persistent presence in the oral cavity. The continued presence of SARS‐CoV‐2 within the oral cavity further weakens mucosal immunity and is associated with various oral pathologies collectively known as oral PASC.

Moreover, the interaction between the oral microbiome and SARS‐CoV‐2 plays a critical role in facilitating viral entry and persistence. We emphasize in the sections below that microbial metabolites and enzymes may enhance viral adherence and penetration into oral keratinocyte cells, bypassing the traditional receptor‐mediated entry pathways [4]. Crucially, the presence of oral bacterial pathogens, particularly in individuals with pre‐existing oral diseases such as periodontitis, can lead to the state of dysbiosis and create a chronic inflammatory state, subsequently dysregulating oral immunity [5, 6, 7]. In turn, a compromised oral immune response can weaken the host's ability to clear the virus effectively, contributing to persistent infection. The extended presence of SARS‐CoV‐2 within the oral mucosa further impairs oral mucosal immunity and associates with a range of oral pathologies–notably dysgeusia, xerostomia, periodontal disease, and mucositis–that characterise oral post‐acute‐sequelae conditions (PASC) and potentially leads to systemic effects (Figure 1) [5, 8]. These dynamics suggest a complex relationship between SARS‐CoV‐2 and the oral microbiome, where microbial activity may exacerbate viral infection and contribute to the suppression of oral mucosal immunity.

**FIGURE 2:**
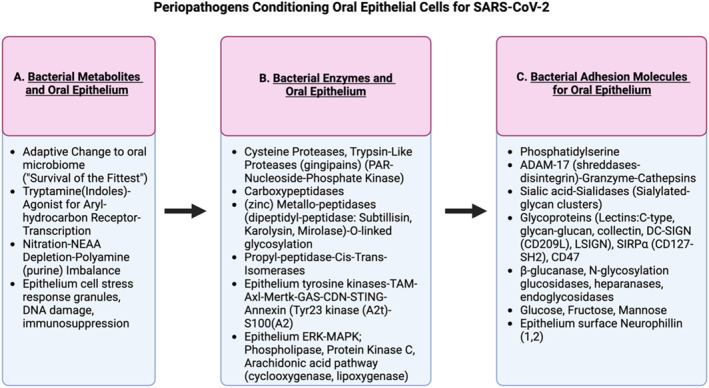
Periodontitis‐driven bacterial dysbiosis in the oral cavity can impair oral mucosal immunity to SARS‐CoV‐2 infection. We propose that periodontal inflammation, supports SARS‐CoV‐2 infection and persistence through activation of various molecular pathways. Here, colonisation by a keystone pathogen, such as *P. gingivalis*, can disrupt the balance of the normal oral microbiome, leading to bacterial dysbiosis. The resulting dysbiosis affects the host's immune response to create a favourable environment for the pathogenic bacteria. These bacteria can alter the regulation of key transcription factors and kinases involved in immune signalling pathways, leading to (1) chronic inflammation due to activation of NFkB and RORγT and (2) attenuated viral clearance due to the manipulation of TBK1, IRF3, and IRF7, which suppresses type I interferon production. In addition, periodontopathogens can modulate host‐derived membrane components, such as phosphatidylserine, in combination with host‐derived metabolites (e.g., AGE, ROS/RNS, and tryptophan) to create a pro‐viral environment, enhancing viral entry and replication. Briefly, periopathogen‐driven chronic inflammation and cell stress leads to the externalisation of phosphatidylserine on the cell surface where it can mimic apoptotic cells, allowing viruses to engage with receptors on phagocytic cells, facilitating viral entry or engulfment; enhanced AGE and ROS/RNS production due to periopathogens causes oxidative damage and weakens the integrity of the oral mucosal barriers and cellular defenses; periopathogen‐driven tryptophan depletion can lead to immunosuppression. Finally, periopathogens can regulate the expression of host enzymes and receptors to increase the number of viral entry receptors and facilitating viral attachment to host cells.

Understanding the interplay between SARS‐CoV‐2 and oral health is critical for developing comprehensive strategies to mitigate the impact of the virus, particularly in vulnerable populations. As the pan‐epidemic continues, it is essential to explore the role of the oral cavity in the pathogenesis of COVID‐19 and its long‐term sequelae. Addressing oral health as part of the broader public health response to COVID‐19 could help reduce the burden of both acute and chronic complications associated with the virus. This review details the important and consequential interactions between SARS‐CoV‐2, the oral microbiome, and the immune system, specifically emphasizing (1) the mechanisms of SARS‐CoV‐2 entry into the oral cavity, (2) the role of poor oral and systemic health, along with oral bacterial pathogens, in facilitating SARS‐CoV‐2 infection and immune clearance deficiency, (4) the contribution of oral fluid dynamics and epithelial turnover in SARS‐CoV‐2 persistence, and (5) the impact of SARS‐CoV‐2 and pre‐existing conditions on oral mucosal immunity. The ultimate goal is to improve patient outcomes and inform future therapeutic interventions.

This review provides a unifying framework for future oral biology studies of SARS‐CoV‐2 infection. We discuss and present specific environmental‐initiated molecular and biochemical features permitting SARS‐CoV‐2 infection in the oral cavity. There is a current lack of information regarding oral mucosal immune responses to changes in the microbiome and the organization of host immune reactivity to viral infections such as SARS‐CoVID‐2. Essential roles for oral microbiome and associated molecular biology need discussion. In this review, we have organised knowledge of oral microbiome, biology, and mucosal immunity to suggest novel biology affecting SARS‐CoV‐2 infection. Attention to this relationship is important because of early and persistent oral pathology and clinically important consequences (e.g., loss of taste, “dry mouth,” loss of smell, and neurologic complications). We further suggest that this review fosters oral mucosal immune studies, suggesting novel opportunities for early diagnosis, diagnosis, treatment, and monitoring of SARS‐CoV‐2 infections and other oral pathogens. We discuss some unique molecular enzymatic biology that affects not only oral immunologic characteristics but also explains oral epithelial susceptibility to this virus, parallelling similar biology in non‐oral tissues and microenvironments.

## 2 Mechanisms of SARS‐CoV‐2 Entry Into the Oral Cavity

Oral infection by SARS‐CoV‐2 is a product of environmental interaction with oral microbiome and mucosa. There are three different sources of information supporting the role of SARS‐CoV‐2 as an oral pathogen. (1) Oral saliva is a well‐documented resource for SARS‐CoV‐2 detection; (2) Different oral tissues (salivary glands, gingival epithelium, tongue papillae) express receptors for SARS‐CoV‐2 adherence and entry; (3) Following a history of COVID‐19, there are clinical presentation of oral disorders designated as “long‐haul” problems. SARS‐CoV‐2 infection is clinically associated with a variety of oral pathologies: dysgeusia (loss of taste), xerostomia (dry mouth), mucositis, opportunistic infections, pulpitis (toothache), periodontal diseases (gum disease: gingivitis, periodontitis), and vesicular‐bullae eruptions [8, 9, 10, 11]. Presentation of signs and symptoms can last for many months following initial infection, suggesting that SARS‐CoV‐2, like other oral pathogens, might reside in the oral cavity for longer than usual and produce a reservoir independent of peripheral or non‐oral sources of infection [12, 13, 14]. These oral conditions suggest persistent viral activity in the oral cavity.

Like other trophic sites, SARS‐CoV‐2 entry in the oral cavity occurs through its interaction with specific receptors expressed on different oral cell types. As mentioned above, ACE2 and TMPRSS (e.g., TMPRSS2, TMPRSS4, TMPRSS11D) are required for adherence and entry into oral epithelial and, in rare occasions, to immune cells. The expression of these receptors varies across oral tissues, with lingual tonsils and lingual surfaces showing higher expression levels than other oral sites, such as the soft or hard palate [15]. This differential receptor expression may influence the severity and persistence of viral infection at specific oral sites.

While SARS‐CoV‐2 entry via receptor‐dependent pathways is known, this review suggests that the oral microbiome provides alternative avenues for SARS‐CoV‐2 infection, bypassing the requirement for activation of ACE2 and TMPRSS2 receptor expressions and precipitating entry into oral keratinocytes. Our proposed bypass pathway suggests that the release of microbial‐derived metabolites and enzymes may facilitate SARS‐CoV‐2 entry into host oral keratinocytes in the gingiva and saliva epithelium preferentially and, to a lesser degree, lingual taste buds associated epithelial papillary cells, both gustatory and accessory. This pathway mimics the requirements for furin‐mediated S protein degradation of S1/S2 domains, which is essential for viral entry. Specifically, this cleavage exposes the CendR motif on the S1 subunit, facilitating membrane fusion and host cell entry. Microbial‐derived serine endopeptidases can mimic endogenous furin, which also induces host cell neuroproteins. One of these neuroproteins is neuropilin‐1, producing a transmembrane signal that aids SARS‐CoV‐2 adherence [3, 16, 17]. In the long term, the activation of both receptor‐dependent and alternative entry pathways may depress viral resistance mechanisms and alter the angiogenic microenvironment, promoting microthrombi formation and tissue ischaemia. These changes could further contribute to developing oral pathologies associated with SARS‐CoV‐2 infection. Understanding these entry mechanisms is essential for developing targeted therapies to mitigate the impact of COVID‐19 on oral health.

## 3 Role of Poor Oral and Systemic Health, Along With Oral Microbiome Dysbiosis, on Viral Infection Susceptibility and Impaired Immune Clearance

Persistent poor oral health depresses oral mucosal immunity, creating vulnerabilities even before SARS‐CoV‐2 exposure. Common opportunistic infections in the gingiva, dental structures, pulp, and oral mucosa exert persistent stress on oral mucosal immunity. This stress results in oral signs and symptoms such as pain, bleeding, dry mouth, altered taste, touch sensations, vesiculobullous eruptions, ulceration, atrophy, fistulation, abscess, cyst, granuloma, and neoplasia growths [18, 19]. Moreover, because poor oral health has an uneven distribution in the oral cavity, severely affected sites can be situated next to relatively healthy ones. This patchwork pattern results in a disorganized mucosal immune response, rendering the oral cavity less capable of resisting SARS‐CoV‐2 infection and managing its associated symptoms (Table 1).

**TABLE 1:** Listed are the head and neck clinical symptoms and pathologies observed in SARS‐CoV‐2 infection.

	Head and neck tissues with clinical and tissue pathology				
		Salivary	Nasal	Gustatory	Brain + central nervous system
SARS‐CoV‐2 receptors and oral cavity related tissue sites of infection					
Tissue‐specific COVID‐19 symptoms	General vascular micro‐thrombo‐embolic events scattered throughout these tissues (salivary, nasal, gustatory, brain + CNS):	Sicca‐xerostomia “dry mouth”, elevation of sIgA months after infection until tissue death.	Anosmia, “Loss of smell”	Hypo‐dysguesia “Loss of taste”	Neurology signs and symptoms (dizzy, confusion, cognitive “foggy brain”)
Protein (enzymes)	Potential roles in SARS‐CoV‐2 pathobiology				
Furin + Pro‐protein convertases	Cleaves spike (S) Protein. N1/N2 domain exposed. PCs exit and assist viral proteins	+ epithelial	+ epithelial	+ epithelial	+ neural tissues‐neuro‐ectoderm
Subtilisin (bacteria)	“Furin‐like” Archaea, bacteria, and eukaryotes	Associated with oral microbiome obtained from saliva	Associated with nasal microbiome ‐nasal secretion	Associated with oral microbiome ‐swab sample	If immuno‐suppression and septicaemia‐ infection ‐CSF fluid (?)
Kexins	“Furin‐like” S8B (fungal)	Associated with commensal fungi‐saliva	If C. *candida* infection	Associated with commensal fungi	If systemic C. *candida* is present
Neuropilin NRP 1/2	Binds to furin‐cleaved substrates	+ epithelial	+ epithelial	+ epithelial	+ neural tissues‐neuro‐ectoderm
TMPRSS2	Epithelial serine protease priming of S2 and increased tropism	+ epithelial	+ epithelial	+ epithelial	‐neural ectoderm tube cells?
Toll‐like‐receptor and intracellular signals	Transmembrane, cell signalling: RIG I/MDAS/MAVS/TRAF3/IRF3/IRF7; by adapters: MyD88/NF‐KB Chemokines: CCL2, CCL3, and CXCL10	+ transmembrane, exosome?	+ epithelial	+ epithelial	+ microglia: neurogenesis, axonal growth and structural plasticity

*Note:* Tissue‐specific COVID‐19‐related head and neck issues include sicca‐xerostomia in the salivary glands, anosmia in the nasal cavity, hypogeusia/dysgeusia in the gustatory system, and neurology signs and symptoms (dizzy, confusion, cognitive “foggy brain”) in the brain and CNS. Scattered throughout the aforementioned tissues are general vascular micro‐thrombo‐embolic events characterised by small and dislodged blood clots. These events can impair blood flow and oxygen delivery, contributing to tissue damage, inflammation, and dysfunction in SARS‐CoV‐2‐infected areas. B. Multiple enzymes are expressed at different head and neck sites, facilitating viral entry and contributing to tissue‐specific manifestations of SARS‐CoV‐2 infection. We highlight that bacterial and fungal‐derived enzymes, such as Subtillisin and Kexins, may further modulate the host immune response to COVID‐19.

While the respiratory tract is the primary site of viral replication, the oral cavity contributes to the viral output, albeit at lower rates. Supporting this concept, approximately similar virus particles are detected in saliva and nasal swabs, indicating that the oral cavity holds a relatively high number of virus copies [20]. However, we note that viral copy number depends upon the variant type of SARS‐CoV‐2 [21, 22]. In a recent report, three target cells were used to address infectivity and replication by SARS‐CoV‐2 variants: one from the lung, one from the intestine, and Green monkey epithelial kidney cells. Results indicated variant infectivity and replication varied depending upon the target, although Omicron may have enhanced activity. Human oral keratinocytes, particularly in gingiva and salivary epithelium, serve as trophic sites for the virus. Co‐infection with other oral pathogens, such as herpesviruses and gram‐positive cocci (e.g., Streptococcus), may further enhance viral infectivity, emphasising the need for more research in this area [23].

### 3.1 Characteristics of Poor Oral Health

Poor oral health in periodontal tissues is defined by several factors:

1. Moderate to severe periodontitis (Stage II‐III)—Probing depths exceeding 4 mm and clinical attachment loss (CAL) of 3–4 mm indicate horizontal bone loss. This can also apply to patients under 30 with over 5 mm of interproximal attachment loss, primarily affecting the first molar and incisors, with no more than two additional teeth involved. While Stage I and early‐Stage II cases generally don't require invasive treatment if tooth loss is minimal, more advanced cases (Stage II‐III) necessitate various periodontal interventions, which signal a poorer prognosis and limited maintenance capability.
2. Oral Disease Classification—Poor oral health can be classified into grades such as Well Maintained, Downhill, or *Extreme Downhill* (grades A, B, or C), which indicate slow, moderate, or rapid disease progression based on post‐treatment needs and tooth loss due to ongoing microbial dysbiosis or inflammation.
3. Associated Oral Mucosal Pathologies—Poor oral health also associates with moderate caries (ICDAS code 2–6) and other oral mucosal pathologies, which are graded based on severity (e.g., oral mucositis graded 1‐3 or using the Oral Mucositis Scale). An example of oral mucosal pathologies is xerostomia (dry mouth), which is assessed on a scale of 0–10, with scores over 4 indicating significant dryness. Overall, previous oral infections and their severity should be considered, as we suggest that natural oral and mucosal immunity may support resistance to SARS‐CoV‐2 and lower the risk of developing PASC [24, 25, 26, 27, 28, 29].

### 3.2 Systemic Implications and Disparities in Oral Health

Poor oral health is more likely among populations with less access to routine oral health care. For instance, in the US, Black and Hispanic communities are reported to exhibit greater susceptibility and mortality to SARS‐CoV‐2 infection [30, 31]. Compared to non‐Hispanic white NHW) people, the COVID‐19‐related death rate of Non‐Hispanic Black and Hispanic people is 1.6 and 1.7 times higher, respectively [32]. This racial disparity is significant because individuals with “poor oral health” may exhibit a loss of viral and bacterial diversity when there is a high titre of SARS‐CoV‐2, suggesting that there may be enhanced competition for attachment sites.

Beyond the oral cavity, various immune, genetic, vascular, and haematologic disorders that also depress antiviral immunity in the mouth. These conditions include familial cyclic neutropenia, dyskeratosis congenita, congenital athymic abnormalities, Thalassaemia, and other haematologic disorders resulting in bone marrow abnormalities, impairing the differentiation of oral immune effector cells, including both lymphocyte and myeloid progenitor populations, such as macrophages and granulocytes [33, 34, 35, 36, 37]. Moreover, these disorders will likely create microbiome dysbiosis not only orally but also in the gut or even extend to the brain, heart, skin, and other tissues, which can further influence oral mucosa in a bidirectional interaction, further hindering clearance of viruses such as SARS‐CoV‐2 and increasing probability for “long‐haul”‐PASC [38, 39, 40, 41].

Even individuals with seemingly “good oral health” may experience SARS‐CoV‐2 infections as “super‐infections” that override their oral mucosal immunity. This relationship contrasts with “poor oral health,” where repeated stress, infection, and abnormal growth increase the risk for depression of oral mucosal immunity, persistent damage to immune surveillance, recognition of antigen, and regulation of oral mucosal repair. Nonetheless, the relationship between compromised oral health and SARS‐CoV‐2 pathogenesis underscores the critical role of oral hygiene and systemic health in mitigating viral risks.

### 3.3 Oral Microbiome Dysbiosis and COVID‐19

**FIGURE 3:**
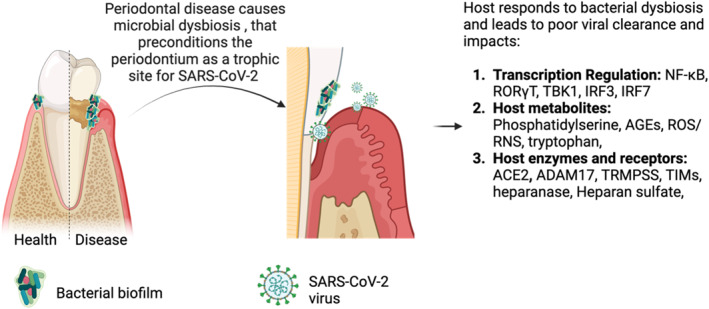
Contributions of pathogenic periodontal bacteria in the pathobiology of periodontal disease. Pathogenic periodontal bacteria produce metabolites and enzymes that may be beneficial for other periodontal microbes, including SARS‐CoV‐2. Several host receptors and adhesion molecules may be required by periodontal bacteria in the same manner as SARS‐CoV‐2, indicating an overlap in the pathways required for the adhesion, entry, and persistence of pathogenic periodontal microbes. For instance, both heparan sulphate (HS) and Heparanase (HPSE) are ubiquitously expressed molecules on a wide range of cell types. Studies suggest that during the initial infection period, the expression of HS proteoglycan is upregulated to facilitate viral entry into host cells [43]. As the virus replicates and matures within the host, the expression and activity of HPSE are concurrently upregulated, promoting the degradation and removal of HS from the cell surface [44]. This dynamic process not only aids in the smooth exit of newly produced virions and spread to other cells but also triggers proinflammatory responses by promoting tissue damage. Oral inflammation driven by microbial dysbiosis can also regulate HS and HPSE expression to ensure that both molecules are present and functionally active at appropriate stages of the viral lifecycle, facilitating efficient infection and viral dissemination.

SARS‐CoV‐2 exposure in individuals with pre‐existing poor oral health (e.g., periodontal disease or dental caries) contributes to establishing a reservoir of persistent oral pathogens, thereby fostering a dysbiotic state [42]. Subsequently, a disrupted oral microbial balance can create an environment conducive to viral persistence and exacerbation of oral diseases. However, there remains a lack of studies addressing the role of pre‐existing and persistent microbiome dysbiosis, a hallmark of periodontitis, as a contributing factor to PASC development. It is important to investigate this gap in knowledge because the oral microbial composition differs drastically between COVID‐19‐positive and COVID‐19‐negative adults. Moreover, changes in oral microbiome diversities at oral sites are individualistic, resulting in vast differences in enzymatic products that produce mimicry of host enzymes. These enzymes facilitate interactions between oral bacteria, SARS‐CoV‐2, and host oral mucosal immune reactions (Figure 1, Table 2), promoting persistent host physiologic reactivity (Figure 3).

**TABLE 2:** Bacteria and fungi release metalloproteases and proteases, contributing to viral infection risks in the oral cavity and the head/neck tissues.

Release of microbial derived metalloproteases assisting with infection risk		
Oral microorganism	Metallo‐peptidase; protease	Sites of head and neck pathology
Pathogenic Periodontal bacteria		
Bacilli *e.g., (Gram positive) Eubacterium nodatum, Slackia exigua, (Gram negative)* aggregatibacter actinomycetemcomitans	Subtilisin	Periodontium, gingiva
Porphyromonas *gingivalis*	Subtilisin, protease IgA1	Periodontium, gingiva
Fusobacteria *nucleatum*	Subtilisin, protease IgA1	Periodontium, gingiva
Streptococcus *gordonii* and S. *pyogenes*	Subtilisin, MMP	Periodontium, gingiva, oropharynx
Neisseria *spp.*	Subtilisin, protease IgA1	Periodontium, gingiva, tongue, buccal mucosa
Capnocytophaga *spp.*	Subtilisin, MMP	Periodontium, gingiva
Treponeum *denticola*	Subtilisin, protease IgA1	Periodontium, gingiva
Actinobacillus *actinomyxcetemcomitans*	Subtilisin, MMP	Periodontium, gingiva
Fungi (genera)		
Candida *albicans*, C. *glabrata*	zinc metallopeptidas, subtilisin/Kexin (kex3 gene)	Disseminate oral mucosa, sinus, pharynx, larynx
Aspergillus *niger*, A. *fumigatus*	Subtilisin/Kexin	“Fungus ball”, sinus, nasal
Actinomyces *spp. Oral taxon 175 F0384*	zinc metalloprotease HtpX	Periodontium, salivary gland
Histoplasmosis *capsulatum*	metallopeptidase	Ocular, palate, disseminates
Blastomycosis *dermatitidis*	zincin metalloprotease	Larynx, oral cavity (palate), neck ear, nasal cavity
Mucomycosis (mucoraceae) *mucor indius*	serine proteases subtilisin	Oropharynx, sinus, rhino orbital, nasal blood clots

*Note:* Microbes enriched during periodontitis release enzymes like subtilisin, zinc metalloproteases, MMPs, and IgA1‐degrading proteases may facilitate interactions between oral tissues and SARS‐CoV‐2, potentially contributing to chronic infections and the development of long‐haul COVID (PASC).

Moreover, prior exposure to PD pathogens may depress the oral immune resistance against SARS‐CoV‐2, although this concept requires further investigation. Nonetheless, multiple lines of evidence show the influence of the oral microbiome on the immune response. For example, periodontitis‐induced dysbiosis can create a chronic inflammatory state in the oral cavity characterised by an increase of pro‐inflammatory markers, IL‐6 and TNFα, as a consequence of Treg function and development [45]. This chronic inflammation can skew the immune response, leading to poor clearance of both viral and bacterial pathogens.

A loss of capacity for clearance of SARS‐CoV‐2 will occur through a loss of normal oral mucosal immune regulatory function due to poor oral health and prior periodontal pathogen exposure, as highlighted below:

1. Loss of antigen presentation and processing by macrophages and tissue‐resident dendritic cells (Langerhans cells) resulting from a problem with the common determinants (B7‐1(CD80)/B7‐2 (CD86/CD28) sites that mediate antigen presentation—This activity requires an associated display of major histocompatibility antigens (major and minor regions) already compromised by poor oral health, potentially by releasing bacterial endopeptidases that degrade peptides before complexing with MHC (Class I/II) sites. Further, through B7 integration with MHC (Class I/II) sites presented by oral epithelial cells. Additional degradation by periodontal pathogen‐derived proteases can further reduce clearance by depressing signalling to elicit pro‐inflammatory cytokines [46, 47]. Supporting this importance of antigen recognition and monitoring of SARS‐CoV‐2 presence was a recent study that observed abatacept, a drug used to block CD80/86, depresses pro‐inflammation caused by COVID‐19 [48].
  A depressed antigen recognition response reduces the clearance of both the SARS‐CoV‐2 virus and other oral pathogens, enhancing the survival of virulent microbes. These microbes display lectins, mitogens, and microbial antigens on their surface and are capable of triggering an adaptive immune response from CD4+ T helper lymphocytes (Th1). SARS‐CoV‐2 infection, especially with persistence and infection of host epithelial cells (gingiva, salivary ductal cells), may alter the intercellular communication by perturbing the production of cargo exosomes or other extracellular multilamellar vesicle structures displaying viral protein. In this context, host miRNAs assist with the evasion of immune recognition through confusing signals for “self and non‐self,” accelerating loss of tolerance [49, 50, 51, 52, 53].
2. Loss of local cytotoxic immune effectors can also occur, particularly with persistent periodontal diseases damaging the distribution of sentinel lymphocytes and their antigenic aggregations (DSLT), resulting in depletion of immune effectors. In an active inflammatory state, these activated lymphocytic cells release granzyme/perforin, enhancing persistent local periodontitis or pyogenic granuloma lesions. Cell loss can also coincide with less control of the microbial release of toxins, microbial‐derived metabolites, and selective survival of pathogens, which can be bacterial, viral, or fungal microorganisms. A relatively rapid loss of immune effectors and change in microenvironment produces changes in the type and amount of antigen (e.g., mitogens and lectins; mannose‐glucose bind‐concanavalin A, peptidoglycans, and lipopolysaccharide) available to immune effectors and host cells. Furthermore, microbial‐derived antigenic change helps to provide a continual re‐education of APCs (e.g., dendritic cells (DC)) [54]. However, after initial stem cell and more mature cell differentiation of CD4+/CD8+, an accumulation of iT_reg,_ other suppressive T cell populations, or anti‐inflammatory macrophage/dendritic cell types (M2) results in less responsive antigen‐interaction (e.g., CD28, CD40, CD44, CD80/CD86, CTLA‐4, etc). An additional consequence, stated above, is the expression of immunosuppressive cytokines (e.g., IL‐10, TGF‐β, adenosine, CD39), all contributing to a reduction in resistance to pathogenic‐virulent microbes. Poor oral health prior to SARS‐COV‐2 exposure accelerates this process, contributing to the blockage of MHC I and II antigen recognition system activity [55].
3. Changes in the microbiome's diversity and balance during dysbiosis disrupt the oral epithelial and immune responses, increasing the likelihood of imbalanced transcriptional receptor systems, notably aryl‐hydrocarbon receptor (ArhR), which drive disease‐promoting T cell differentiation into iTregs [56]. Microbial‐derived proteases exacerbate this process by degrading critical immune components like secretory IgA, promoting Th17 accumulation, and enhancing the loss of immune tolerance.

Table 1 provides additional descriptive details for microbial‐derived enzymes and likely host response factors (Table 1). We emphasize a role for toll‐like receptors (TLR) signalling, a pathogen pattern recognition (PPR) pathway that triggers innate inflammation during SARS‐CoV‐2 infection by oral epithelial cells and similar cell types identified by saliva (epithelial‐myoepithelial cells), nasal fluid (pseudo‐stratified, columnar epithelium), and taste bud (gustatory cells/support epithelial cells). The TLR system produces a differential signal from the cell membrane and endosomal‐lysosomal membrane that results in the activation of specific TLR components, allowing for bacterial versus viral adhesion signalling to help a critical element for poor oral mucosal clearance response [57]. Below, we describe three mechanisms by which oral dysbiosis can influence the severity of SARS‐CoV‐2 pathogenesis:

1. Synthesis of Metabolic Derivatives—Oral pathogens synthesise metabolites or precursor molecules that regulate host biochemical pathways, utilising essential and non‐essential amino acids (NEAA). These metabolic derivatives contribute to host oxidation and energy metabolism, affecting the mitochondrion, cellular organelles, and genomic activity. Furthermore, microbial expression of 15′‐cis‐phytoene synthases facilitates the synthesis of RORγT receptors and ArhR activity, driving Th17 differentiation. At the same time, this process promotes the expression of IL‐22, which enhances the production of antimicrobial peptides (AMPs) that protect the oral mucosa.
  Additionally, the natural tendency of oral microorganisms to coaggregate with one another particularly during oral disease, enhances the metabolic release of reactive oxygen/nitrogen species (ROS/RNS), such as nitric oxide. In turn, nitric oxide serves a signal for nitrosation change in the oral cavity, which could serve as a relatively easy marker for monitoring SARS‐CoV‐2 presence under dysbiotic conditions [58, 59, 60, 61, 62].
  Moreover, these metabolic by‐products of coaggregation contribute to the accumulation of advanced glycosylation as end products‐receptor (AGE/RAGE) indicates an energy regulation and oxidative control function by host oral epithelial cells as they interact with immune effectors [61, 62, 63]. This system's significance helps to develop a loss of tolerance linked to the capacity to clear oral pathogens. However, this process may be influenced by the release of a dipeptidyl peptidase IV mimic that has a diabetogenic function through enhanced incretins [64]. This mechanism links severe periodontal disease to systemic conditions such as diabetes and its role as comorbidity factor for COVID‐19‐PASC [65].
  In addition to these metabolic effects, microbes and inflammation provide ligands that activate retinoid receptor activation and PPARs (e.g., RAR‐RXR and PPAR‐RXR). These receptors recruit coactivators and corepressors to regulate complex gene expression programs. Crucially, activation of PPARγ pathway suppresses Th17 differentiation, reducing the risk of immune tolerance loss [66]. Notably, 112 oral bacterial species—including *Neisseria m*acacae ATCC, *Neisseria sicca*, *Prevotella intermedia*, *Pseudomonas stutzeri*, and *Rhodobacter capsulatus*—possess the enzymatic capacity to produce retinoids. Additionally, these bacteria synthesise butyrate via arginine pathways, which activates PPARγ and induces autophagy in immune cells, contributing to immune modulation and maintaining mucosal integrity [67]. These processes highlight the intricate relationship between microbial metabolites and host immune regulation.
  Building on these regulatory mechanisms, fat metabolites are a rapid energy source supporting ligand adherence for peroxisome‐proliferator activator receptor, PPAR, and other receptors such as TRPA1 and ETS‐1 transcription complex signals. This is particularly relevant in an environment enriched in oral pathogens (e.g., periodontal pathogens) from pre‐existing oral diseases. Here, pre‐existing oral diseases provide a select expression of lipid (e.g., LPS) and phosphorylated ethanolamine, serine, and choline along with endopeptidases, including proteases (granzyme, cysteine), serine proteases (legumain, cathepsins, gingipains, trypsin‐like proteases) and metallopeptidases orchestrating signal manipulation required to govern normal homoeostatic cell physiology. Together, this composition deranges membrane response to mimic endogenous host enzymes that mediate cell response to the microenvironment. One important endogenous enzyme is furin, a pro‐protein convertase (Subtillisin, Karilysin, and fungal Kexin) (Table 2.). Additionally, proteases influence the host's common determinant response network. e.g., microbial‐derived proteases can activate CD47 to affect acute immune reactivity (e.g., macrophages, PMNs, complement, NK) and cell‐mediated differentiation (CD8+).
  Furthermore, glycation and glucan products issued from periodontal co‐aggregating pathogen complexes (e.g., P *ginigvalis*, F. *nucleatum*, S. *gordonii,* T *denticola,* Tannerella *forsythia*) trigger T cell immunoglobulin mucin domains (TIMs). This alternative endosomal‐lysosomal cascade for activation swamps normal signals, providing a new avenue for SARS‐CoV‐2 endocytosis [52]. Some newly activated receptors are tyrosine‐type receptors linked to Tam‐Axl‐STING‐CDN, a tyrosine kinase, calcium‐dependent transcriptional response system associated with epithelial cells and phagocytes during innate inflammation and T cell differentiation. These receptors also mediate the removal of apoptotic bodies of cells and the differentiation of T cell effectors for antigen processing and differentiation following the binding of phosphatidylserine (PS)—a membrane phospholipid externalised on host cells during apoptosis—and glucan—a polysaccharide derived from microbial cell walls [68, 69]. Collectively, these metabolic and molecular interactions underscore the complex interplay between oral microbial communities and host immune responses, emphasising their critical role in shaping susceptibility to COVID‐19.
2. Release of Proteolytic Enzymes—In the previous section, we described how oral pathogens can assist the entry of SARS‐CoV‐2 into oral tissues by releasing proteolytic enzymes. Here, we contend that microbial endopeptidases, often characterised by zinc‐dependent metallo‐domains, play a crucial role in tissue breakdown and immune modulation.
  Building on this, specific pathogens like *P. gingivalis* further illustrate the role of bacterial proteolytic enzymes in SARS‐CoV‐2 pathogenesis. P. gingivalis releases proteases, which include gingipains (arginine (RgpA, RgpB) and lysine‐specific (Kgp) enzymes. Upon co‐aggregation with other oral pathogens, *P. gingivalis* triggers a cascade of intracellular periopathogen interactions, ultimately releasing legumain, a cysteine asparagine endopeptidase. Legumain aids in binding to membrane molecular hydrophilic active sites while attracting glycoproteins and sialylated complexes like L‐SIGN/DC or even ACE2 [70, 71]. ACE2 functions as an ectoenzyme of zinc metallo‐peptidase with carboxypeptidase (di‐carboxy‐peptidyl‐peptidase) activities; additional microbial‐derived zinc metallo‐peptidases could also potentiate these activities.
  We noted the significant role of lectin‐glycoprotein in aiding SARS‐CoV‐2 adherence. Furthermore, we stressed that periopathogen presence facilitates the release of protease (e.g., ADAM17) to provide SARS‐CoV‐2 S protein, allowing ACE2 attachment (e.g., 1‐2 receptors μm^−2^). In addition, as the periopathogen undergoes vacuolation and enters the oral epithelial cell, ADAM17 helps to push the pathogen into the cell through the induction of autophagy [71, 72, 73]. This increases stress and membrane damage, resulting in DAMP signalling. In addition, ADAMs cause the shedding of cytokine receptors from immune effectors. This activity results in the accumulation of soluble forms of cytokines producing clinical signs (e.g., IL‐1 and fever) while others (e.g., IL‐6) stimulate B lymphocyte population growth and antibody synthesis. MMPs and ADAM17 precursors also often require serine endopeptidase‐proprotein convertase and furin to activate cleavage sites of S1/S2 for adherence to SARS‐CoV‐2. Higher ADAM17 activity from inflamed gingiva suggests an impaired immune activity, enhancing the loss of tolerance and promoting poor clearance activities of viruses and bacterial pathogen persistence [74]. Simultaneously, other metallopeptidases contribute to this processing, including cathepsins, granzyme, and Annexin‐A2, further propelling pathogens inward into the cell and reducing resistance to clear SARS‐CoV‐2. For example, cathepsins, the most abundant lysosomal degradative enzymes, include serine (A and G), aspartic (D and E), and cysteine proteases (B, C, F, H, K, L, O, S, W, and Z). In general, cathepsins focus on proteolysis activity to regulate endosomal attachments. In this context, they regulate endosomal TLR9, IL‐1β, and TNF‐α expressions required to generate a pro‐inflammatory host response to promote clearance, possibly facilitating SARS‐CoV‐2 adherence and endocytic entry [75].
  ADAM‐17 protease activity works cooperatively with additional signals generated through surface molecules of pathogens. For example, PS surface display by periopathogens induces ADAM‐17, which sheds T‐cell immunoglobulin‐like (TIM) proteins [68, 69, 74]. TIMs can interact with PS displayed on the surface of periopathogens. This TIM‐PS interaction depresses immune resistance and releases additional endoproteases, including legumain (cysteine protease), subtilisin, (MMP) karilysin, and (MMP) microlaser. These MPPs increase probable adherence for SARS‐CoV‐2 but also depress the innate complement and cell immune resistance [76].
3. Activation of Host Signalling Pathways—Microbial surface molecules engage host cell membrane signals, triggering transcriptional responses (e.g., Signal Transducer and Activator of Transcription (STATs), Nuclear factor of activated T‐cells (NFATs), Interferon regulatory factors (IRFs), etc.) organise cellular processes, immune function and resistance to oral pathogens. These responses support general housekeeping and maintain cell integrity. However, a shift from symbiosis to dysbiosis, driven by an overabundance of oral pathogens, disrupts these pathways, creating a disease microenvironment characterised by inappropriate signalling. This dysregulation ultimately contributes to oral pathologies and weakened mucosal immunity against viruses (Figure 2).
  Microbial surface molecules such as bacterial endotoxin lipopolysaccharide (LPS) or other membrane components (e.g., phosphatidylserine and phosphatidylcholine) in combination with lectins‐glycoproteins (e.g., glucan‐glycans) trigger host oral mucosal epithelial and immune effectors populations to produce a pathogen pattern recognition response (PRR). This signalling activates various defence mechanisms, such as DAMPs, PAMPs, and AMPs, and stimulates tyrosine receptors like Annexin2‐S100A2 to mediate immune responses. In addition, glycation activities from periodontal disease pathogens drive advanced glycation end product (AGE) levels, further dysregulating membrane signals and host cell biology and immune responses [77, 78, 79, 80, 81, 82].
  Overall, the interplay between SARS‐CoV‐2, oral disease, and the oral microbiome underscores the critical role of oral health in modulating viral infection dynamics and highlights the need for targeted interventions to mitigate the risk of severe oral and systemic complications.

## 4 Role of Oral Fluid Dynamics and Epithelial Turnover in SARS‐CoV‐2 Persistence

Oral fluid dynamics play a critical role in regulating oral mucosal immunity and influencing the persistence of SARS‐CoV‐2 in the oral cavity. Viral reservoirs host SARS‐CoV‐2 and other persistent viruses (e.g., herpesviruses and human papillomavirus), fostering competition for adherence sites and highlighting the importance of host secretions (e.g., saliva, nasal fluids, tears) in modulating these interactions. The composition of these salivary, nasal secretions, and tears are important because they introduce antimicrobial enzymes and proteins—such as amylases, albumin, agglutinins, defensins‐cathelicidins‐lysozyme‐lactoferrin, immunoglobulins, cytokines, gastin‐carbonic, anhydrase‐pH modifiers, mucins, glycol‐lipoproteins, proline‐rich proteins, histatins, cystatins—which influence the metabolism and integrity of oral tissues and immune responses [83, 84, 85, 86, 87, 88, 89].

This complex array of chemicals from saliva and gingival crevicular fluid composition identifies the need to examine oral fluids to uncover factors influencing biofilm and microbiome diversity, richness, and evenness of microbial distribution (e.g., viruses, bacteria, and fungi). Therefore, in addition to the microbiome‐derived metabolites and enzymes and subsequent host‐synthesised metabolites and enzymes, there are salivary‐ and GCF‐associated molecules that may be useful in disclosing oral biology relationships and indicate a rich source of targets to detect SARS‐CoV‐2 infection and risk for “long‐COVID” while initiating an oral inflammatory axis to the brain, gut, heart, kidney, surface epithelium, bone, or immune cells or other tissues.

Surface adhesion molecules displayed on the cell or endosomal membranes (e.g., proteoglycan, glucosaminoglycans, lipoproteins, glycan, etc.) further influence viral attachment and persistence of SARS‐CoV‐2 as oral fluids bathe tissues. However, a dynamic set of membrane proteins mediate viral adherence to surface molecules from microbes and microenvironment, fostering organization. One group of host factors are functionally related neuropeptides called soluble *N‐*ethylmaleimide‐sensitive factor attachment protein receptors (SNAREs), which regulate fusion pore sites and membrane layering required for endocytotic activity, as noted with SARS‐CoV‐2 [90].

The quantity and quality of oral fluids change, possibly influencing the adhesion and persistence of SARS‐CoV‐2 in oral and non‐oral issues and their clinical manifestations, such as xerostomia, dysgeusia, gingivitis, mucositis, and swallowing with difficulty (i.e., dysphagia). Still, also postural orthostatic tachycardia (POTs), “foggy‐brain” and chronic fatigue syndrome‐like disorder, CFS, anosmia, and enteritis‐enterocolitis are reported without the presence of respiratory disorders but recorded using C‐reactive protein values [91, 92, 93, 94, 95].

Epithelial turnover rates further modulate viral adhesion and infection dynamics. The oral keratinised surfaces exfoliate at different rates, which could further affect the adhesion of SARS‐CoV‐2. For example, ortho‐keratinised mucosa present on hard palate surfaces has a 24‐day epithelial cell turnover, the dorsum tongue, the site of taste buds, has a 35‐day turnover, while the attached (mucogingival line) gingival vestibule has a 41‐day exfoliation period. In comparison, shorter turnover periods are para‐keratinised‐nonkeratinised mucosal surfaces. These are marginal‐sulcus gingiva, with a turnover of 16–18 days, or non‐keratinised oropharynx with 16–18 days, hypopharynx with 18 days, and base of the tongue with 16–18 days of turnover. The number of turnover days is significant because an extended period indicates a slower and more differentiated epithelium with thicker keratin protection. In contrast, a shorter turnover time exposes less differentiated oral epithelium, with a thinned keratin protective layer to the oral environment (e.g., enzymes, pH change, redox change, metabolites) and damage from SARS‐CoV‐2. Supporting this point, immunohistochemical expression for ACE2 and TMPRSS2 is shown to be generally higher in salivary > gingival > tongue > buccal > palate tissues. SARS‐CoV‐2, a ssRNA, oral infection is likely a product of select tropic interactions that model microenvironments along with conditioning physiological changes in oral epithelium as is shown by the DNA viruses, Herpesviruses exemplified by cytomegalovirus (HHV‐5), Epstein Barr virus (HHV‐4) or Herpes Simplex Virus 1 (HHV‐1) while accompanying other RNA viruses from Coxsackievirus Group A [3, 96, 97, 98]. In conclusion, the interplay between oral fluid composition, epithelial turnover, and viral reservoirs provides a nuanced understanding of how SARS‐CoV‐2 persists and propagates within the oral cavity, contributing to both local and systemic manifestations.

## 5 Impact of SARS‐CoV‐2 Infection on Oral Mucosal Immunity and the Oral Microbiome

The following section details how SARS‐CoV‐2 infection affects site‐specific immune and microbiome changes, compromising clearance of other opportunistic pathogens. The loss of oral mucosal immunity, particularly against SARS‐CoV‐2, exacerbates viral persistence, leading to systemic complications such as “long‐haul Covid” or PASC. We contend that the development of “long‐haul Covid” or (PASC) is the accumulation with a high probability for continual low‐level replication infecting or reinfecting tissues. This contention is based upon the fact that RT‐qPCR detected SARS‐CoV‐2 RNA in saliva weeks after infection [99].

This situation would create local site‐specific microbiome and immune reactivity changes, leading to site‐specific impairments in the clearance of SARS‐CoV‐2 and potentially other viruses. Figure 3 and Table 3 describe potential pathways for impaired oral mucosa immunity during oral SARS‐CoV‐2 infection (Figure 3; Table 3). Additionally, we anticipate a dysregulation in immune effector activities, involving both innate and adaptive responses (Table 3). Host‐generated oral immune responses, depicted in Figure 3, reflect a depleted immune cell reserve—characterised by anergy or exhaustion and diminished differentiation capacity—resulting in reduced antiviral defence (Figure 3). Table 1 explains oral health consequences related to the site of infection (e.g., loss of taste, loss of salivary flow, poor oral health) (Table 1).

**TABLE 3:** SARS‐CoV‐2 Infection has been shown to impair both the innate and adaptive immune responses, including in the oral cavity.

Anticipated general exhaustion of oral immune responses to SARS‐CoV‐2	
Innate immunity	Effect
Complement	Oral mucosa contains capacity to elicit C1‐9. Convertase complexes
NK cells	Reduction in number and function, reduces chemokines
Macrophage (M1/M2)	Activation release pro‐inflammatory cytokines, modifies chemokine: CCL2, CCL3, and CXCL10, modified by granulocyte activities
Dendritic cells/Langerhans cells	Part of monocyte population antigen presentation to IELs and γ/δ T cells cross talk dampens lysosomal degradation increase virion release
Granulocytes	
Eosinophils	Initial increase then decrease oral eosinophils number and functional loss of viral clearance as replication continues but may increase antigen presentation to Th2 with further enhancement of innate reactivity
Mast cells/Basophils	Loss of eosinophil regulation; assist in micro vascular inflammation
Polymorphonuclear cells	Cytokine storm enhances PMNs association with vascular haemorrhagic leakage after initial viral exposure
Intraepithelial lymphocytes (IELs (γ/δ T cells)	Loss of communication and activity with decrease in numbers with DCs/Langerhans cells with severe infection
Adaptive immunity	Innate to adaptive immune response; mucosa and vascular damage in oral cavity and at distant sites (brain, lung, heart, kidney, intestines)
CD3+/CD4+ (Th1/Th2) (γ/δ T cells)	Leucopenia; loss of cytotoxicity (fas‐apoptosis) are expected particularly in severe cases with loss of memory T cells (CD45RO+)and risk for mortality
CD4+/CD25+/Foxp3+ (T_reg_)	Lowered levels of T_reg_ which initiates CD8+ c1 activity promoting tissue and cell damages expected
CD4+ (Th17)	Increase in number and function increase autoantibody with loss of tolerance associated with dysbiotic microbiome
CD8+ (CD8+c1)	Cytotoxicity cell target (apoptosis) with viral infection; parallel tissue damage (severe infection). Reduced number, activity over time also reduces tumour surveillance and tumour cytotoxicity

*Note:* (A) Affected innate immune responses include: (1) chronic activation of the complement system, which can continuously produce pro‐inflammatory anaphylatoxins (C3a and C5a), impairing resolution of inflammation and resulting in tissue damage, (2) reduced number and impaired function of natural killer (NK) cells, delaying the innate immune response to SARS‐CoV‐2 infection, (3) prolonged pro‐inflammatory activation due to a shift of macrophage polarization to a highly pro‐inflammatory phenotype (M1‐like macrophages) and the subsequent release of pro‐inflammatory cytokines, (4) impaired antigen presentation to IELs (γ/δ T cells), resulting in reduced immune system activation and dampened lysosomal degradation; dampened lysosomal degradation diminishes the immune system's ability to breakdown viral particles and thereby contributing to virion release from infected host cells and (5) dysregulated immune responses by granulocytes, including an eventual decrease in eosinophil numbers and subsequent impairment of viral clearance. With the loss of eosinophil regulation, viral replication progresses, and mast cells and basophils contribute to microvascular inflammation and exacerbate periodontal disease. Moreover, excessive activation of PMNs heavily contributes to the cytokine storm observed in severe COVID‐19, promoting vascular leakage and haemorrhage after initial viral exposure. Finally, the reduced communication of IELs (γ/δ T cells), which display innate‐like and adaptive‐like characteristics, with dendritic and Langerhans cells delays or weakens the T cell response, resulting in a more robust viral replication in the oral cavity. (B) Affected adaptive immune responses include: (1) observed leucopenia (reduction of WBCs) in severe cases of COVID‐19. Depletion of CD3+/CD4+ Th1/Th2 and γ/δ T cells and memory T cells (CD45RO+) impairs long‐term immunity and weakens viral recognition, (2) reduction of CD4+/CD25+/Foxp3+ Treg cells leading to uncontrolled CD8+ T cells, contributing to excessive tissue damage and cell death, (3) increased CD4+ Th17 cell activity, promoting autoantibody production and loss of immune tolerance; loss of immune tolerance is also associated with a dysbiotic microbiome, (4) excessive CD8+ (cytotoxic T cells) associates with increased tissue damage due to excessive apoptosis of infected cells. Once CD8+ T cells become exhausted in severe cases of COVID‐19, tumour surveillance and tumour cytotoxicity are compromised, potentially affecting long‐term immune function, and increasing cancer risk.

An expected consequence of SARS‐CoV‐2 infection is the compromised clearance of other opportunistic oral pathogens (i.e., bacteria, fungi, and viruses), suggesting a broader issue in microbial regulation. As mentioned above, pre‐existing poor oral health compounds this depressed immune clearance, suppressing oral mucosal immunity and facilitating enhanced viral tropism and replication.

In addition, we expect that SARS‐CoV‐2 infection affects oral microbiome metabolism for essential and non‐essential amino acids. These amino acids are required to maintain survival for some bacteria in the oral microbiome (Figure 3). For example, L‐citrulline to L‐arginine and nitrosylation create nitric acid (NO). NO triggers host epithelial cell inducible nitric acid synthase (iNOS). NO affects blood flow, induces apoptosis, and loss of NK function. In addition, other biochemical reactions are expected: methionine yielding cysteine and contributing to glutathione reductase (Phase II) transsulfuration activity contributing to improper polyamine linked to poor erythroid and myeloid cell differentiation (e.g., RBC, monocytes, lymphocytes), with a reduction of B complex influence on transulfuration and SAM‐methylation creating faulty DNA repair, ultimately encouraging SARS‐CoV‐2 entry into epithelial cells [100].

Furthermore, SARS‐CoV‐2 presence may help explain the loss of commensal bacteria and the accumulation of pathogenic bacteria. This dysbiotic microbiome produces tryptophan/indoleamine metabolites, which trigger the ArHR pathway producing immunosuppression. Prior immunosuppression (e.g., poor oral health) may also have a profound effect, causing loss of adaptive immune‐lymphocyte function from antigen‐presenting cells.

Oral health deterioration can occur through increases in reactive oxygen substances (ROS), which increases the risk for oral mucosal pathologies as identified by oral PASC (e.g., mucositis, gingivitis, periodontitis, sialadenitis, changes in taste buds, taste sensitivity, specificity). ROS generation during SARS‐CoV‐2 infection likely triggers a reduction of cellular protective ROS quenching systems generated by phase II enzymes but supports trans‐sulfuration via cysteine and methionine synthesis. The reflection of ROS is also defined by the related activity of DNA repair with oral mucosal turnover compensating for cell autophagy and tissue damage [101]. Together, the crosstalk between host and oral microbiome‐derived factors actively contributes to SARS‐CoV‐2 tropism, viral persistence, and loss of antiviral immunity, precipitating various clinical manifestations in the oral cavity. These “long haul” PASCs are end‐clinical manifestations with complex origins, as described above, in oral and non‐oral tissues.

The tongue, responsive to dietary changes, also serves as a key interface for microbiome‐host metabolic changes. Unlike other sites, it must integrate oral microbiome metabolism with taste papilla gustatory cells and supportive accessory cells such as Tuft cells. Tufts cells are exceptionally responsive to neuropeptides and neural transmitters. Many bacteria in this microenvironment use zinc to synthesise metallopeptidases, while depriving cells of zinc contributes to sensing response difficulties. Accessory activities of high‐affinity transport systems, mobilisation of zinc ions from cell reservoirs, producing zinc‐independent paralogs, and shifting metabolic pathways are susceptible to zinc‐directed therapy to reverse taste dysfunction—one of these metabolic pathways regulation of fat metabolites, specifically fatty acids products. Oxidative fatty acid products are ligands for receptors such as ArhR and transient‐potential receptor calcium‐channel system (TRPA1) in taste buds. Activated receptors produce inflammatory responses through a calcium‐dependent transcription activation, such as the ETS‐1 complex. ACE2 expression reportedly correlated significantly with genes upregulated by the transcription factor ETS‐1, regulating oxidative stress. This process overlaps with NRF2‐KEEP1 expression, which is responsive to ArhR transcriptional activity [102].

Ligand‐dependent ArhR requires transcription control because this pathway generates the production of immunosuppressive immune products and produces DNA damage through phase I enzymes (e.g., cytochrome P_450_: 1A1,1A2, etc.). A ligand‐dependent cullin‐based E3 ligase activity, ubiquitination, and (26S) proteasome activation are implemented. These ligands include lipid inflammatory molecules, indoleamines, arachidonic acid‐related molecules, and 6‐Formylindolo [3,2‐b]carbazole (FICZ). Various inhibitors, including ROS and PAMPs, can block the FICZ/AHR/CYP1A1 feedback loop. Indoleamine 2,3 dioxygenase derived from the bacterial tryptamine pathway in conjunction with tryptophan synthesis in DCs causes depletion of amino acids (NEAA). Increasing the presence of Indoles and triggering the arginine‐citrulline pathway can enhance the survival of T cells iT_reg_. This cell will synthesise adenosine from ATP because of CD39 and CD73 activation to reduce antigen presentation responses, hindering Th1 and Th2 responses to antigens and inhibiting immune response [103].

This degradation system controls the dimerisation required for ArhR binding to Arnt as part of ligand complex activation. ArhR activity is regulated by Cullin‐based E3 ligase and ubiquitination mediated by oxidative state through hypoxia‐inducible factor (HIF‐1) [104]. HIF‐1 competes with ArhR to bind to Arnt. However, HIF‐1 is also a product of TMPRSS transmembrane proteases modified by phosphatase and tensin homologue (PTEN)/mTOR pathway expressed in oral epithelial cells. Therefore, the induction of HIF‐1, which targeted ArhR functionally, resulted in Arnt binding. This interaction also involves transcription regulatory factors (STAT‐3, Aiolos, ETA‐1) blocking IL‐2 and depressing T cell differentiation for Th1, which will secure a shift towards the Th17 phenotype and loss of tolerance. As noted above, ArhR production of iT_reg_ lymphocytes increases immunosuppression (e.g., TGF‐β, IL‐10, adenosine, CD39) and reduces cell cytotoxicity, promoting the loss of tolerance [105, 106].

To sum up, an unresolved pathogenic SARS‐COV‐2 activity promoted by oral microbial pathogens enhances the induction of iT_regs_ that will consume IL‐2 through CD25, a required cytokine for proper CD4+ T cell differentiation, to increase the synthesis of immunosuppressive cytokines (IL‐10, IL‐35, TGF‐β). This will, in turn, accelerate the exhaustion and depletion of T cells through the release of perforin and granzyme and enhance pyroptosis and apoptosis through FAS/FASL. Enhanced MHC disruption will occur, precipitating antigen recognition and processing errors and release of inhibitory factors such as lymphocyte activation gene‐3 (LAG3)—MHC II; activation of programme‐cell death‐1 (PD‐1/PD‐ligand), suppressing cytotoxic T‐lymphocyte antigen (CD152, CTLA‐4), which decreases B7 (1/2) CD80/CD86 function through a reduction in maturation and blockage. The product is an exhausted and poorly functioning immune response tilted towards loss of tolerance. This view is supported by the presence of PASC in both oral and non‐oral settings.

## 6 Summary

The presence of poor oral health prior to SARS‐CoV‐2 infection is likely to accentuate oral signs and symptoms. Poor oral health is recognisable through oral microbiome changes in metabolism and increased pathogenic microbes, causing a more disorganized and less efficient host oral mucosal immune clearance. The resulting immune response is likely inefficient to sustain antiviral resistance to SARS‐CoV‐2 infection. Furthermore, oral mucosal immune defects enhance the risk of additional oral viral infections from DNA and RNA viruses, accompanying physiologic and metabolic impairment and enhancing bidirectional inflammatory axes between oral and non‐oral tissues, promoting risk for PASC. Multiple microbial infections could explain prolonged oral signs and symptoms exacerbating persistent oral health conditions such as periodontal and endodontic diseases, and dissemination of these perturbations to non‐oral sites, including the brain, heart, kidney skin, and gut.

## Author Contributions

Joel Schwartz and Afsar Naqvi conceptualised the review. Kristelle Capistrano, Heba Hussain, Avin Hafedi, Joel Schwartz and Afsar Naqvi prepared the figures and compiled the tables. Joel Schwartz, Kristelle Capistrano, Deepak Shukla, and Afsar Naqvi wrote and edited the manuscript.

## Conflicts of Interest

The authors declare no conflicts of interest.

## Data Availability

The authors have nothing to report.
